# The Effect of Beta-Alanine versus Alkaline Agent Supplementation Combined with Branched-Chain Amino Acids and Creatine Malate in Highly-Trained Sprinters and Endurance Athletes: A Randomized Double-Blind Crossover Study

**DOI:** 10.3390/nu11091961

**Published:** 2019-08-21

**Authors:** Krzysztof Durkalec-Michalski, Krzysztof Kusy, Monika Ciekot-Sołtysiak, Jacek Zieliński

**Affiliations:** 1 Institute of Human Nutrition and Dietetics, Poznan University of Life Sciences, 60-624 Poznań, Poland; 2 Department of Food and Nutrition, Poznan University of Physical Education, 61-871 Poznań, Poland; 3 Department of Athletics, Strength and Conditioning, Poznan University of Physical Education, 61-871 Poznań, Poland

**Keywords:** supplements, training support, sport, physical capacity, body composition, biochemical markers

## Abstract

The study aimed to verify the effect of intra- (beta-alanine—BA) versus extra- (alkaline agents—ALK) cellular buffering agent supplementation, combined with customarily used branched-chain amino acids (BCAAs) and creatine malate (TCM) treatment in natural training conditions. Thirty-one elite athletes (11 sprinters and 20 endurance athletes) participated in the study. Eight-week randomized double-blind, crossover, combined supplementation with BA-ALKpla_BCAA&TCM_ and ALK-BApla_BCAA&TCM_ was implemented. In the course of the experiment, body composition, aerobic capacity, and selected blood markers were assayed. After BA-ALKpla_BCAA&TCM_ supplementation, total fat-free mass increased in sprinters (*p* = 0.009). No other differences were found in body composition, respiratory parameters, aerobic capacity, blood lactate concentration, and hematological indices after BA-ALKpla_BCAA&TCM_/ALK-BApla_BCAA&TCM_ supplementation. The maximum post-exercise blood ammonia (NH_3_) concentration decreased in both groups after BA-ALKpla_BCAA&TCM_ supplementation (endurance, *p* = 0.002; sprint, *p* < 0.0001). Also, lower NH_3_ concentrations were observed in endurance athletes in the post-exercise recovery period. The results of our study indicate that combined BCAA, TCM, and BA supplementation is more effective than combined BCAA, TCM and ALK supplementation for an increase in fat-free mass and exercise adaptation, but not for aerobic capacity improvement. Besides, it seems that specific exercise stimuli and the training status are key factors affecting exercise performance, even in athletes using efficient supplementation.

## 1. Introduction

In sports practice, it is often difficult to cover the increased athlete’s need for energy and nutrients through only a standard diet. For this reason, adequate supplementation is an important factor supporting the training process. From the multitude of supplements, athletes mostly use preparations such as branched-chain amino acids (BCAAs), creatine (Cr), β-alanine (βA) and sodium bicarbonate (SB). These supplements are associated with several mechanisms that affect the metabolism and adaptation of the human body to physical exercise.

BCAAs can significantly affect the stimulation of the mammalian target of the rapamycin (mTOR) kinase pathway and thus stimulate the synthesis of muscle proteins, as well as have anti-catabolic activity, related to inhibition of muscle protein proteolysis [1,2,3]. The above mechanisms may explain the observed effect of BCAA supply on the exercise-induced muscle damage, pain, fatigue or injury reduction, and the acceleration of fat-free mass or strength recovery [1,2,4,5]. Some additional benefits may also be observed for endurance (aerobic) exercise because BCAAs have ergogenic potential and may affect energy sources metabolism [6]. Some papers indicated the possible relationship between BCAAs and the reduction of central fatigue, which is associated with the synthesis of serotonin and facilitated by reduced BCAA/tryptophan ratio [7,8]. Such data show the importance of BCAA supplementation during long-term training periods. Ultimately, however, the effectiveness of BCAAs in athletes may also depend on the availability of other essential amino acids [5,9].

Cr is one of the most commonly used supplements to stimulate phosphocreatine (PCr) synthesis, increase PCr concentration, and enhance the effectiveness of adenosine triphosphate (ATP) resynthesis in muscle tissue [5,10,11]. Cr may also support high-intensity exercise and upregulate anabolic signals provided by exercise stimuli [5]. These mechanisms allow Cr to affect the development of muscle strength and power, exercise capacity in single and repeated sprints, and support lean body mass (LBM) increase, which is particularly relevant to strength and speed–strength sport disciplines [5,10,11]. The Cr supplementation is therefore beneficial to the competitors of various sports, although its supply in endurance disciplines does not seem justified [12]. However, some observations suggested a Cr-related increase in the effectiveness of muscle glycogen resynthesis, aerobic metabolism (via improvement of ATP shuttling from mitochondria), anaerobic threshold, and exercise tolerance, accompanied by a decrease in blood lactate accumulation [10,11,13]. This indicates some benefits of Cr supplementation also in endurance athletes. Cr may also have the potential to counteract injury and support treatment and rehabilitation [5,10,11].

A serious problem is muscle acidification, closely related to intense training and competition. Acidification leads to muscle fatigue due to the competition of H^+^ with calcium ions for the troponin binding site, suppression of phosphocreatine resynthesis and oxidative phosphorylation, inhibition of key enzymes of the glycolytic pathway (such as glycogen phosphorylase and phosphofructokinase), and decrease in the mitochondrial energy production in muscle cells (due to reduced mitochondrial matrix-cell cytoplasm proton gradient) [14,15,16]. 

Given the above-mentioned exercise-induced homeostasis disturbances, βA supplementation may be an effective counteraction [5,17,18,19,20]. Carnosine, synthesized from βA, is the main intracellular factor that buffers the excess of H^+^ ions (generated in the process of glycolysis in muscle fibers) and thus suppresses the muscle acidification [5,17,18,19,20]. Furthermore, the potential impact of βA on muscle sensitivity and efficiency of Ca^2+^ release, neuromuscular fatigue suppression, antioxidant and anti-glycation effects, and detoxification of exercise-induced metabolites can also be beneficial in athletes [17,20,21]. It seems that the βA supply is particularly efficient in efforts lasting 60–240 s, e.g., in speed–strength disciplines in which the development of speed, power, and endurance strength is most important [5,15,17,19,21,22,23]. However, further assessment of this compound requires further research in disciplines in which exercise lasting <1 min (e.g., sprint) or >7 min (e.g., endurance disciplines) is dominant, especially in the case of longer supplementation periods under real training conditions.

A growing interest in ergogenic support is also seen in the case of alkalizing agents (ALK) such as SB, sodium citrate, and sodium/calcium lactate. Their impact on blood alkalosis and increase in extracellular buffer capacity are described in the literature [5,15,24]. The supply of alkalizing compounds counteracts the exercise-induced muscle acidification by H^+^ binding and greater efflux of H^+^ and lactate from muscle, thus ensuring sustained muscle contractility during exercise [5,15,25,26]. The benefits of the extracellular metabolic alkalosis can also be associated with membrane depolarization, mitochondrial adaptations, and acceleration of glycogenolysis, which may enhance exercise performance [27,28,29]. The above mechanisms seem to explain the effectiveness of ALK observed in the studies on efforts lasting ~1–4 min, but the results obtained for the exercise of longer duration are inconclusive [5,15,30]. It was observed that SB supplementation increased performance, speed, and muscle power. It also reduced the time to reach peak power, increased total mechanical work, endurance strength, and improved sport-specific exercise abilities in speed–strength disciplines and multiple bouts of exercise [5,15,31,32,33,34,35].

Our study aimed to assess the effect of the supplementation of intra- (BA) versus extra- (ALK) cellular buffering agents, combined with customarily ingested BCAAs and Cr in real training conditions in highly-trained athletes. The above aspects of the most popular supplements often did not reflect the chronic supplementation in highly-trained athletes who use dietary supplements much more frequently than their non-elite counterparts and often concurrently use many preparations [26,36,37,38]. Moreover, evidence-based supplementation protocols are often isolated from other ergogenic aids or habitual training. These issues include the additive, interactive, or counteractive effect of combined use of different supplements and the repeated use of a supplement during one event or individual responsiveness [26,38]. Also, although there is no evidence of a direct effect of BA and ALK supplementation on body composition and aerobic capacity, the reduction of exercise-induced muscle acidification may affect muscle efficiency during training. This could affect training-induced aerobic adaptation and body composition. According to our practical observations, we hypothesize that chronic BA and ALK treatment will support the training process in terms of the favorable effect on exercise adaptation and body composition.

## 2. Materials and Methods 

### 2.1. Participants

**Figure 1:**
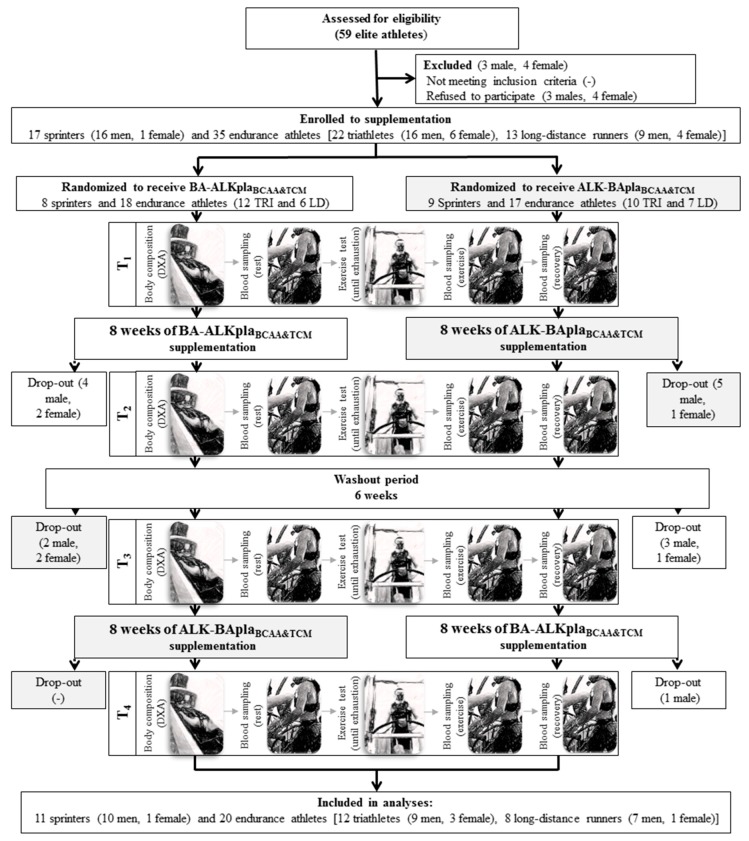
The flow chart of the study design. Abbreviations: ALK—Alkaline agents, BA—Beta-Alanine Carno Rush, BCAA—branched-chain amino acids, DXA—Dual X-ray Absorptiometry, LD—long-distance runners, pla—placebo, TCM—creatine malate, and TRI—triathletes.

Fifty-two participants (17 sprinters, 22 triathletes, and 13 long-distance runners) were initially enrolled in this study. Eventually, 31 subjects completed the entire study protocol and were included in the analysis: 11 sprinters (10 men, 1 woman) and 20 endurance athletes (12 triathletes (9 men, 3 women) and 8 long-distance runners (7 men, 1 woman)) (Figure 1, Table 1). The participants were professionally trained athletes and members of the Polish National Team. The inclusion criteria were age from 18 to 35 years, good health, a valid medical certificate confirming the athlete’s ability to practice competitive sport, and at least 5 years of training experience. The gender-related impact of the study was assumed negligible because the crossover design of our study allowed the participants to be their own control group. Furthermore, under real training conditions, the studied female athletes consumed the same supplements as male athletes. Also, there is a lack of evidence in the literature that the applied supplementation protocol was dependent on the sex of the subjects. Moreover, the assumption was also not to change athletes’ nutrition and eating habits during the course of the study.

Exclusion criteria were less than 6 training sessions per week, cigarette smoking, use of banned stimulants, alcohol consumption more than 1–2 drinks per week, and dietary supplements use beyond the recommendation of the authors of this study. For females, additional exclusion criteria were being pregnant or planning to become pregnant during the study.

Before the study, the athletes customarily used preparations such as carbohydrates, BCAA, and Cr. However, they did not use any intra- (BA) or extra- (ALK) cellular buffering agents. Moreover, during the study period, all athletes declared that they did not introduce any changes in their lifestyles, especially nutrition, and that they did not use any medications and supplements with potential ergogenic effects, other than those supplied by the authors of this study. The dietary and workout records were collected during the run-in period of the study (Table 1, Table S1). Furthermore, every second week, the consultations with a dietitian and a member of the research team were held, which ensured that the athletes did not change their dietary habits and training mode during the whole supplementation period. 

The primary recruitment strategy was to contact the national team coaches. They enabled the identification and confirmation of required inclusion criteria declared by the participants (such as training experience and the number of training sessions per week). They also supported the compliance of supplementation with the study design.

The project was approved by the Ethics Committee at the Poznan University of Medical Sciences (143/15 of 5 February 2015) and was performed according to the ethical standards laid down in the Declaration of Helsinki. Each subject was informed of the testing procedure, purpose, and risks of the study and submitted her/his written consent to participate. The study was conducted from December 2015 to December 2016. The study complies with the CONSORT Statement for randomized trials, as shown in Figure 1.

### 2.2. Experimental Design

#### 2.2.1. Supplementation Characteristics

The effect of supplementation was assessed in a randomized, crossover double-blind trial (Figure 1). The use of combined supplementation was intended to reflect the real conditions in which athletes use these preparations and to assess the synergistic effect of supplements on the exercise capacity of athletes. Upon being qualified to the experiment, the athletes were subjected to a randomization procedure and assigned either to (1) the group receiving an BA-ALKpla_BCAA&TCM_ (Beta-Alanine Carno Rush Mega Tabs^®^, BCAA Mega Caps^®^, TCM Mega Caps^®^, and placebo (PLA) instead of Alkagen™) or (2) to the group receiving ALK-BApla_BCAA&TCM_ (Alkagen™, BCAA Mega Caps^®^, TCM Mega Caps^®^, and PLA instead of Beta-Alanine Carno Rush Mega Tabs^®^) preparations. The random allocation and assigning participants to the supplementation with a specific set of preparations was performed by an impartial scientist who was not a member of the research team. The experimental procedure included 8 weeks of BA-ALKpla_BCAA&TCM_ and ALK-BApla_BCAA&TCM_ supplementation. After this period, a 6-week washout period was introduced [20,22]. The next step, after the washout period, was the crossover exchange of the preparations between the groups.

During each period of supplementation, the athletes were given the equivalent quantitative doses of 0.2 g·kg_FFM_^−1^ branched-chain amino acids (BCAA Mega Caps^®^; 1100 mg·cap^−1^) and 0.05 g·kg_FFM_^−1^ creatine malate (TCM Mega Caps^®^; 1100 mg·cap^−1^). Depending on the period of study, the following preparations were also administered in the BA-ALKpla_BCAA&TCM_ group: 5 g·day^−1^ beta-alanine (Beta-Alanine Carno Rush Mega Tabs^®^, containing βA (1000 mg·cap^−1^), sodium citrate (150 mg·cap^−1^), 1-histidine HCl (500 mg·cap^−1^), and vitamin B_6_ (0.35 mg·cap^−1^)) and PLA (maltodextrin) imitating Alkagen^®^ alkalizing formulation. Concurrently, the following preparations were administers in the ALK-BApla_BCAA&TCM_ group: 0.2 g·kg_FFM_^−1^ Alkagen^®^ alkalizing preparation [containing SB (375 mg·cap^−1^), potassium bicarbonate (375 mg·cap^−1^), calcium phosphate (150 mg·cap^−1^), potassium citrate (125 mg·cap^−1^), magnesium citrate (125 mg·cap^−1^), calcium citrate (90 mg·cap^−1^), magnesium oxide (30 mg·cap^−1^), and zinc (0.375 mg·cap^−1^)] and a PLA (maltodextrin instead of a beta-alanine). The preparations were administered in four split doses: upon waking, 45 min before a training session, immediately after a training session, and before sleep.

All products and PLA preparations were prepared by Olimp Laboratories (Dębica, Poland), who have experience in manufacturing sports supplements according to high-quality production standards. Preparations were labeled using special codes, making it impossible to identify them and assign the same preparation twice to the same subject. 

#### 2.2.2. Study Visits

The athletes visited the laboratory four times (T_1–4_, Figure 1). At each visit, body mass and composition were measured, and an exercise test was performed. All tests were conducted in the Human Movement Laboratory “_L_A_B_THLETICS” in the Department of Athletics, Strength and Conditioning at the Poznan University of Physical Education. The subjects were instructed not to participate in any high-intensity or long-duration training session at least 24 h before testing. The tests were performed in the morning, 3 h after a light breakfast (no coffee or tea). Before the exercise test, subjects underwent body composition analysis. Afterward, an incremental treadmill exercise test until volitional exhaustion was performed. During all examinations, room temperature remained at 20–21 °C. The athletes were familiar with the incremental exercise test until volitional exhaustion because they participated in some previous studies and routine examinations.

Anthropometrics and Body Composition. Body mass (kg) and height (cm) were measured using a digital stadiometer (SECA 285, Hamburg, Germany). Body mass index (BMI) was calculated by dividing body mass by height squared. The Dual X-ray Absorptiometry (DXA) method, utilizing the Lunar Prodigy Pro device (GE Healthcare, Madison, WI, USA) and enCORE v. 16 SP1 software, were used for body composition analysis. During the DXA examination, subjects only wore their undergarments, without jewelry and metal objects to minimize measurement error. The test was carried out according to the standardized scanning protocol as recommended by Nana et al. [39].

Respiratory Parameters. An incremental exercise test until volitional exhaustion on a mechanical treadmill (H/P Cosmos Pulsar, Sports & Medical, Nussdorf-Traunstein, Germany) was performed to determine the maximal oxygen uptake (VO_2max_). The initial speed was set at 4 km·h^−1^ and increased after 3 min to 8 km·h^−1^. After that point, treadmill speed increased progressively by 2 km·h^−1^ every 3 min until volitional exhaustion. Once the speed of 10 km·h^−1^ was reached, blood samples were drawn from the athlete at the end of each 3-min stage. Respiratory parameters were measured (breath by breath) using the Metamax 3B R2ergospirometer (Cortex Biophysik, Leipzig, Germany) and analyzed using the MetasoftStudio v. 5.1.0 software package (Cortex Biophysik, Leipzig, Germany). Heart rate (HR) was monitored with the Polar Bluetooth Smart H6 heart rate monitor (Polar Electro Oy, Kempele, Finland). 

Blood Sampling. Subjects wore a catheter (BD Venflon Pro 1.3 × 32 mm, Helsingborg, Sweden) patent with isotonic saline (0.9% NaCl), through which blood was drawn from the antecubital vein. Blood samples were collected at rest, immediately after exercise (maximum exertion, max), and 5, 20, and 30 min into the post-exercise recovery period (R5, R20, and R30, respectively). Then, a 2.7 mL blood sample was taken into two monovettes (S-Monovette 2.7 mL KE, Sarstedt, Nümbrecht, Germany), one with a lithium anticoagulant (heparin) and another containing an anticoagulant (EDTA).

Lactate and Ammonia. The Biosen C-line analyzer (EKF diagnostic GmbH, Barleben, Germany) was used to measure lactate levels. To determine lactate concentration, 20 µL of whole blood was placed into a capillary. The measurement accuracy (CV) was 1.5% for 12 mmol·L^−1^ concentration. To determine the ammonia level, the PocketChem BA PA-4140 analyzer (Arkay, Kyoto, Japan) was used. To perform a measurement using a testing strip (Ammonia Test Kit II, Arkay, Kyoto, Japan), 20 µL of blood was placed using a pipette. The measuring range was 8‒285 µmol·L^−1^ and the CV was 2.3%.

Hematological measurements. The hematological analysis was carried out on the 18-parametric automated hematology Mythic^®^18 analyzer (Orphée, Geneva, Switzerland) using 10 µL of blood.

Diet evaluation. The customary diet was assessed by recording all consumed foodstuffs, dishes, and beverages, specifying home measures and weight (using “The photo album of food products and dishes”). Dietary records were performed during the run-in phase of the study. Study dieticians gave instructions to each participant individually on how to complete the diaries. The participants recorded time and the amount of food and beverages consumed at each meal. The energy and nutrient intake from diaries were calculated using Dietetyk-2 software (JuMar, Poznań, Poland). A dietician reviewed and discussed the diary with each participant. Furthermore, every second week, consultations with a dietitian were held, which allowed monitoring athletes’ adherence to a customary diet and proper compliance with the supplementation protocol.

#### 2.2.3. Statistical Analysis

The randomization was performed in a stratified design using fat-free mass (FFM) as a prognostic variable [20,32,40,41]. The results were presented as the means ± standard deviation (and 95% confidence intervals). The normality of data distribution was tested using the Shapiro–Wilk test. If the distribution was not normal, the Box-Cox transformation was applied. Data were analyzed using the repeated-measures analysis of variance (ANOVA) with the inclusion of experimental supplementation order (BA-ALKpla_BCAA&TCM_ first or ALK-BApla_BCAA&TCM_ first), which allowed for the elimination of the potential carry-over effect. The analysis also included factors independent of time, i.e., discipline (sprint/endurance) × treatment (BA-ALKpla_BCAA&TCM_/ALK-BApla_BCAA&TCM_) × period (Pre/Post). Post hoc analysis was done using the Bonferroni correction. When the sphericity assumption was violated, the Greenhouse–Geisser and the Huynh–Feldt corrections were performed. The sample size was estimated a priori, assuming at least a medium effect size. Assuming α-level of 0.05 and statistical power (1–β) of 0.80, it was calculated that at least 13 participants would be needed to detect a significant change or differences in lactate and plasma purine metabolite concentration (G*Power; Heinrich-Heine-Universität Düsseldorf, Germany). To compare anthropometric data, training experience, and diet characteristics, *t*-tests for independent samples or Mann–Whitney U tests were performed, depending on data distribution (normal or not normal, respectively). Statistical significance was set at *p* < 0.05 and data were analyzed using the Statistica 12 software (StatSoft Inc., Tulsa, OK, USA).

## 3. Results

### 3.1. Baseline Characteristics

From among fifty-two participants, 15 males and six females did not complete the study due to participation in training camps, changes in training schedule, not adhering to the protocol, or withdrawal without explanation (Figure 1). There were no differences between the groups (sprinters vs. endurance athletes) in terms of training experience, absolute maximal oxygen uptake at baseline, and diet characteristics (energy, protein, fat, and carbohydrate intake) in the course of the study (Table 1). The only differences (*p* < 0.05) concerned body height, body mass, energy (in kcal/kg^−1^) and carbohydrate intake (in g/day^−1^).

### 3.2. Body Composition

**Figure 2:**
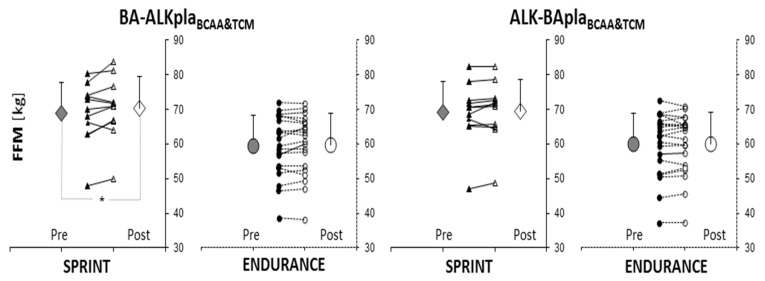
Changes in fat-free mass before and after supplementation. Abbreviations: ALK—Alkaline agents, BA—Beta-Alanine Carno Rush, BCAA—branched-chain amino acids, FFM—fat-free mass, pla–placebo, and TCM—creatine malate. *: significant difference between “Pre” and “Post” values (*p* < 0.01).

Data on body composition are included in the Table S2. In the sprint group, total fat-free mass increased after BA-ALKpla_BCAA&TCM_ supplementation (+2.4% FFM; Pre: 68.7 ± 8.9 kg vs. Post: 70.4 ± 9.1 kg, *p* = 0.009) (Figure 2). No significant differences between the sport disciplines were found before and after supplementation.

### 3.3. Cardiorespiratory Indices before and during the Incremental Exercise Test

There were no significant changes in the level of cardiorespiratory and aerobic capacity indices after the BA-ALKpla_BCAA&TCM_/ALK-BApla_BCAA&TCM_ interventions, neither in the sprint nor endurance group (Table 2). 

### 3.4. Biochemical Blood Markers

#### 3.4.1. Resting and Post-Exercise Blood Biochemical Marker Concentrations

**Figure 3:**
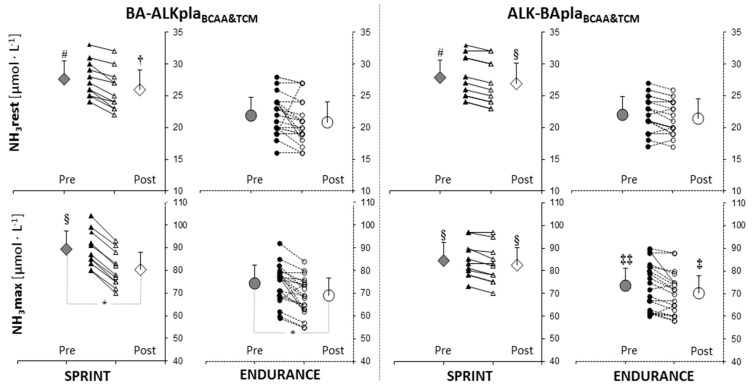
Resting and maximum post-exercise blood ammonia (NH_3_) concentration before and after supplementation. *: significant difference between “Pre” and “Post” values (*p* < 0.001); #,§: significantly different from the endurance group for all periods (# *p* < 0.001, § *p* < 0.01; †: significantly different from the endurance group for BA-ALKpla_BCAA&TCM_ periods and “Post” ALK-BApla_BCAA&TCM_ (*p* < 0.05); ‡: significantly different from the endurance group for “Pre” BA-ALKpla_BCAA&TCM_ period (*p* < 0.05); ‡‡: significantly different from the endurance group for “Post” BA-ALKpla_BCAA&TCM_ period (*p* < 0.05).

In both groups, the maximum post-exercise blood ammonia (NH_3_) concentration decreased after BA-ALKpla_BCAA&TCM_ supplementation by ~10.1% in the sprint group (Pre: 89.4 ± 8.0 µmol·L^−1^ vs. Post: 80.4 ± 7.7 µmol·L^−1^, *p* = 0.000038) and by ~7.3% in the endurance group (Pre: 74.4 ± 8.4 µmol·L^−1^ vs. Post: 68.9 ± 8.3 µmol·L^−1^, *p* = 0.001512) (Figure 3). In the endurance group, NH_3_max concentration was lower after BA-ALKpla_BCAA&TCM_ supplementation than before ALK-BApla_BCAA&TCM_ (*p* < 0.05), whereas NH_3_max concentration after ALK-BApla_BCAA&TCM_ treatment was lower than before BA-ALKpla_BCAA&TCM_ supplementation (*p* < 0.05) (Figure 3). In sprinters, NH_3_max was significantly higher than in the endurance group before and after ALK-BApla_BCAA&TCM_, and before BA-ALKpla_BCAA&TCM_ treatment, but not after BA-ALKpla_BCAA&TCM_. Resting NH_3_ was also higher in sprinters than in endurance athletes, regardless of the supplementation mode. Resting and post-exercise lactate concentration and the level of hematological indices were not significantly different between the groups after supplementation (Table S3).

#### 3.4.2. Blood Biochemical Marker Concentrations during the Post-Exercise Recovery Period

**Figure 4:**
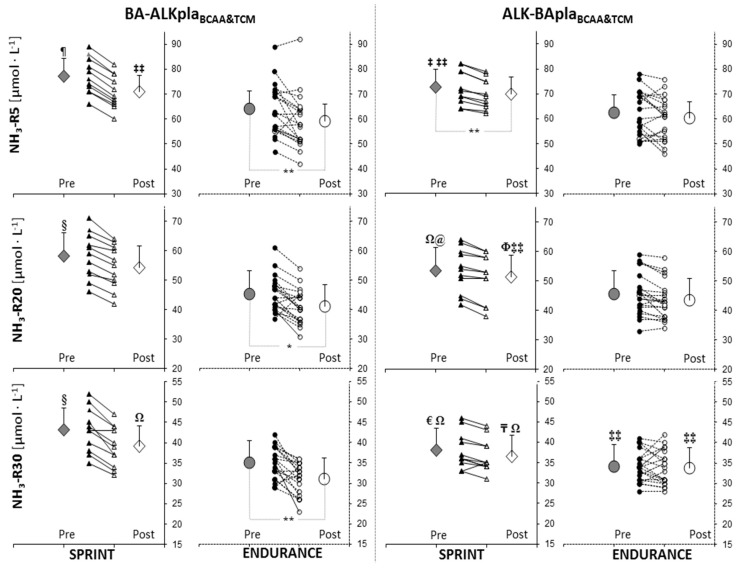
Blood ammonia (NH_3_) concentration after 5, 20, and 30 min of the post-exercise recovery period (R5, R20, and R30) before and after supplementation. *,**: significant difference between “Pre” and “Post” values (* *p* < 0.001, ** *p* < 0.05); §,¶: significantly different from the endurance group for all periods (§ *p* < 0.01, *p* < 0.05); ‡: significantly different from the endurance group for “Pre” BA-ALKpla_BCAA&TCM_ period (*p* < 0.05); Ω,‡‡: significantly different from the endurance group for “Post” BA-ALKpla_BCAA&TCM_ period (Ω *p* < 0.001, ‡‡*p* < 0.05); @: significantly different from the endurance group for “Post” ALK-BApla_BCAA&TCM_ period (*p* < 0.05); Φ,₸,€: significantly different from the sprint group for “Pre” BA-ALKpla_BCAA&TCM_ period (Φ *p* < 0.01; ₸ *p* < 0.001; € *p* < 0.05).

In endurance athletes after BA-ALKpla_BCAA&TCM_ supplementation, a reduction in NH_3_ after 5, 20, and 30 min of post-exercise recovery was observed (NH_3_-R5: −7.7%; Pre: 64.2 ± 10.4 µmol·L^−1^ vs. Post: 59.3 ± 11.0 µmol·L^−1^, *p* = 0.034917; NH_3_-R20: −9.3%; Pre: 45.4 ± 6.1 µmol·L^−1^ vs. Post: 41.2 ± 5.8 µmol·L^−1^, *p* = 0.000548; NH_3_-R30: −11.1%; Pre: 35.1 ± 3.9 µmol·L^−1^ vs. Post: 31.2 ± 3.9 µmol·L^−1^, *p* = 0.000155). In the sprint group, the NH_3_ concentration was lower compared to the baseline after 5 min of post-exercise recovery (−3.6%; Pre: 72.5 ± 6.8 µmol·L^−1^ vs. Post: 69.9 ± 6.0 µmol·L^−1^, *p* < 0.05) only after ALK-BApla_BCAA&TCM_ (Figure 4).

Before BA-ALKpla_BCAA&TCM_ supplementation, NH_3_ concentrations in the whole 30-min recovery period were higher in sprinters compared to all endurance athletes. After the BA-ALKpla_BCAA&TCM_ treatment, sprinters had significantly higher NH_3_ concentrations than endurance athletes at the 5th and 30th (but no 20th) minute of the post-exercise recovery. Besides, in the endurance group, the values for “Pre” BA-ALKpla_BCAA&TCM_ period NH_3_-R5 were lower than in sprinters for the “Pre” ALK-BApla_BCAA&TCM_ period. In the endurance group, “Post” BA-ALKpla_BCAA&TCM_ NH_3_ concentrations in each restitution period were lower than in the “Pre” ALK-BApla_BCAA&TCM_ period in sprinters. Moreover, differences in the endurance group were observed at NH_3_-R30 (“Post” BA-ALKpla_BCAA&TCM_ vs. “Pre” (*p* < 0.05) and “Post” (*p* < 0.05) ALK-BApla_BCAA&TCM_ period).

## 4. Discussion

We found that in elite athletes, in real training conditions, the combined supplementation with (1) customarily used supplements (BCAAs and Cr) and (2) intra- (BA) or extra- (ALK) cellular buffering agents increased fat-free mass and improved some biochemical indices of exercise adaptation. However, the administered supplementation did not affect the aerobic capacity, lactate concentration, nor the concentrations of hematological markers. The observed favorable changes seem to relate mainly to speed and strength, rather than endurance exercise, which suggests that the training specificity is a key factor. Although it was not possible to fully monitor the actual training load during the study period, it can be assumed that the increase in intra-cellular buffer capacity, associated with βA supplementation, allowed for better exercise performance and adaptation. This could explain both the increase in FFM and the reduction in NH_3_ concentration in the sprint group. Smith et al. [40] and Hoffman et al. [41,42] showed that βA supplementation allowed for a stronger exercise stimulus (higher exercise intensity and volume), which could explain parallel changes in LBM. Moreover, apart from the effect on the exercising muscle, it was possible that intramuscular acidosis also negatively affected protein synthesis and proteolysis processes [43,44]. For this reason, the intra-cellular buffer capacity by acid-base balance regulation may favorably affect muscle protein balance kinetics and exercise adaptation also through this mechanism.

In the case of body and fat mass (FM), our observations are consistent with those of other authors analyzing the impact of βA intake. In sports science students (not experienced in resistance training), 10 weeks of resistance training combined with βA did not result in any significant change in body mass, % body fat, whole-body strength, isokinetic force production, and upper arm curl test compared to PLA [42]. Similarly, in untrained collegiate females undergoing an 8-week resistance training, LBM improvement and FM reduction were observed, with no significant changes for PLA in both groups. These observations seem to be valid for untrained participants subjected to regular sports training [43]. In recreationally active women subjected to high-intensity interval training, body mass did not change significantly after supplementing or a 3-week training [23]. However, similar as in our study, only the βA group significantly improved LBM from baseline to mid-testing (*p* = 0.011) [45]. Also, in recreationally active females, no significant differences in body mass and body composition were reported [44].

However, the above studies did not include professional athletes. After a 28-day βA supplementation in female masters athletes (cyclist), no change in body composition was found [46]. However, in strength–power athletes (collegiate football players at least 2 years of resistance training experience), an increase in LBM and a decrease in %FM were observed after a 10-week resistance training program concurrent with combined βA and Cr supplementation, which was not shown for supplementation with Cr or PLA alone [47]. In another 8-week study including trained football players and collegiate wrestlers supplemented with βA (in blend formula with N-acetyl l-cysteine, alpha-lipoic acid, and vitamin E), no significant changes in the FM and LBM were observed in spite of some favorable changes in these indicators under PLA conditions [48].

It should be noted that although relatively few studies verified the effect βA has on body composition, we did not find any studies in which the intake of alkaline agents during a training period would be combined with body composition monitoring. For this reason, our work fills a gap in this area and indicates that the chronic supply of extracellular buffering agents does not affect body composition in sprinters and endurance athletes.

In our study, we did not observe any changes in respiratory and aerobic capacity indices after the BA-ALKpla_BCAA&TCM_/ALK-BApla_BCAA&TCM_ interventions, regardless of the training regime (sprint vs. endurance). It seems that this may have resulted from the elite sports level and high physical capacity of the tested athletes. In such individuals, it is difficult to obtain a significant effect of physiological adaptation in a relatively short time (~8 weeks). In support of our results, Baguet et al. [21] showed that a 4-week βA supplementation reduced acidosis during high-intensity cycling in physically active male students, however, without affecting oxygen uptake kinetics and oxygen deficit. It should be emphasized that those participants were not involved in regular organized training/competition before and during the study. We believe that the specificity of the exercise stimuli associated with the training regime can impact on the effectiveness of the agents supporting buffer capacity. This view is also supported by Smith et al. [45] who demonstrated that in recreationally active men βA supplementation combined with high-intensity interval training (3 HIIT workouts per week, 3 weeks at 90–110% of VO_2_peak and then 3 weeks at 115% of VO_2_peak) resulted in significant improvements in VO_2_peak (~11.9%), time to fatigue (~18.6%), and total work (~124.8%).

Moreover, it is worth noting that the combination of BA with other supplements often used by athletes may advantageously affect aerobic capacity. Combined supplementation with βA and Cr resulted in a significant increase in VO_2_, power output at lactate threshold (LT), and percent of VO_2_peak at VT [49]. The combination of these agents was more advantageous than supplying βA or Cr alone. However, only untrained males, not enrolled in any planned training procedure during supplementation, participated in that study, which explains the lack of changes in VO_2_ peak. This can be supported by similar observations in untrained women, in which the time to reach the VT and the time to exhaustion (Texh) during incremental cycling increased, however, without an effect on maximal aerobic power [50]. Similarly, no significant change in VO_2_max and Texh after an 8-week resistance training was shown [43]. Moreover, in recreationally active females, the implementation of HIIT training (three times a week) combined with βA supplementation led to an increase in VO_2_peak in both βA and PLA groups [51]. There was no significant change in the control group and between βA and PLA at any time point. Similarly, Smith et al. [23] detailed aerobic capacity improvement in moderately trained women in both βA- and PLA-supplemented groups, but without any significant increase in VT and Texh during the graded exercise test. In the study of Kresta et al. [44], no aerobic adaptation changes were observed in moderately active female supplemented with βA, Cr, or βA + Cr. It seems, therefore, that further research in this field is needed.

It should be underlined that data on the effect of other ALK supplementation on aerobic capacity strengthening is scarce. There is also a lack of research on long-term ALK supplementation in combination with other supplements. In noncycling trained males, Higgins et al. [52] showed that one-time pre-exercise SB, caffeine (CAF), and SB + CAF intake did not affect VO_2_, VE, and respiratory exchange ratio (RER) during high-intensity cycling and the only differences were found in the case of greater HR after the combined supplementation of SB and CAF or CAF supplementation in comparison to PLA. Similar changes in SB and PLA group were observed in taekwondo athletes (in the case of VO_2_ and HR in three consecutive exercise rounds) [53], and in boxers (without changes in HRmax after SB supplementation) [35]. Similarly, in a double-blind crossover PLA-controlled study including 15 moderately active men, no change was found in VO_2_ and Texh during supramaximal efforts after acute SB supplementation, with only minor changes in VCO_2_ and VE [54]. It also seems that the pre-exercise SB treatment, despite lowering the exercise-induced acidification, does not affect VO_2_ slow component in the tests at 90% VO_2_max, as demonstrated in a study including professional road cyclist [55]. Similarly, in elite male BMX cyclists, despite significant blood alkalosis, no significant SB effects on oxygen uptake, carbon dioxide excretion, HR, and VE were observed, except for some changes in HR variability [56]. Moreover, progressive chronic SB supplementation among CrossFit-trained participants increased the time to VT, the workload at VT, and heart rate at VT, however with no significant change in VO_2_max [31]. However, although SB ingestion did not affect VO_2_, VE, and RER, it could significantly accelerate post-exercise acid-base balance recovery and Texh [57].

It seems that a one-off supply of extra-cellular buffering agents should not be expected to significantly affect exercise adaptation. This would be difficult to achieve, especially in highly-trained individuals. However, when planning the training in practice, it should be noted that the pre-exercise alkalosis can counteract the VO_2_ decrease related to mild acidosis, probably as a result of changes in minute ventilation and muscle acid-base status [58]. In our study, a long-term supplementation was implemented, which may have contributed to greater exercise capability. However, training loads were not monitored, nor was cardiorespiratory response analyzed immediately after ingestion of alkaline agents. Therefore, we cannot assess their effectiveness in this context.

In our study, no supplementation-related changes in resting and post-exercise lactate and hematological indices concentrations have been demonstrated. It can be assumed that the long-term supplementation in trained athletes (e.g., as a result of homeostatic mechanisms and/or body’s adaptation) may reduce the influence of some ergogenic substances on changes in blood biochemical markers [41]. However, it must be stressed that the NH_3_ concentration in the blood was an indication that the applied supplementation had some beneficial effects. It was found that regardless of the specificity of the training and typical differences between disciplines (higher in sprinters than endurance athletes—*p* < 0.01), the maximum post-exercise blood NH_3_ concentration decreased in both groups after BA-ALKpla_BCAA&TCM_ supplementation by ~7.3% (endurance, *p* = 0.0015) and ~10.1% (sprint, *p* < 0.0001). The benefits were also observed in the post-exercise recovery period. These findings may be particularly important because previous observations indicated that blood NH_3_ concentration may be used as a sensitive blood marker for the exercise-related metabolic response in the competitive sprint- and endurance-trained athletes during an incremental exercise [59]. The change in NH_3_ concentration over time can be regulated especially by amino acid and adenosine monophosphate (AMP) deamination, but also by exercise specificity, ATP, PCr, glycogen stores utilization/depletion, and nutritional/supplementation support with amino acids or carbohydrates, which interfere with NH_3_ metabolism [60,61,62,63,64]. For this reason, the advantageous effect we observed (lower NH_3_ concentration) may indicate less exercise-induced homeostasis disturbances, probably associated with the suppression of amino acid and AMP deamination, which seems to prove better adaptation to exercise [60,65,66]. Although it was not investigated in our work, it is possible that the combined supplementation also promotes buffering potential limiting ammonemia, probably by increasing NH_3_ clearance via the increased promotion of urea cycle intermediates.

Despite the importance of this topic, relatively little scientific data is available on the connection between supplementation and NH_3_ concentration in athletes. Some mixture of amino- and keto-acids can influence on post-exercise ammonemia suppression [67,68]. Moreover, in male cyclists after a 2 h cycling session followed by a maximum test, a significant increase (by ∼70%) in NH_3_ concentration in the PLA group was observed, with no change of this indicator in the group supplemented with combined amino acids and keto analogs [69]. It was also demonstrated that exercise-induced hyperammonemia is suppressed by both arginine (combat sports athletes) [60] and glutamine (professional football players) [62] chronic supplementation. Also, ergogenic treatment with carbohydrate, glutamine, or their combined supplementation limited hyperammonemia in high-level endurance athletes during prolonged strenuous exercise in a field situation [63].

Some observations suggest that that BCAAs may increase NH_3_ concentration, especially during exercise [3,70,71]. Some differences in the NH_3_ concentration, however not statistically significant, were observed in juvenile athletes (before, at the end of maximal-intensity rowing exercise test, and during recovery period) between BCAA-, l-glutamine-, and PLA-treated groups [72]. In young swimmers supplemented with combined BCAA, citrulline, and arginine, NH_3_ concentration remained unchanged with significantly higher plasma BCAA concentrations and lower tryptophan/BCAA ratio [73]. In runners, no significant difference was observed in peak blood NH_3_ between the alkaline (sodium citrate) and PLA treatment [74]. However, the duration of exercise could be too short (30‒40 s) to induce substantial changes.

It is also worth mentioning that a lower increase in NH_3_ concentration can also be linked to a slower rate of glycolysis and glycogenolysis, especially in trained muscle [65,75]. Therefore, even maintaining exercise capacity (not only an increase) can prove a lower physiological cost of muscle work. Albeit, it would require further confirmation. However, a lower NH_3_ concentration can inhibit the adverse effect of ammonia on protein synthesis and BCAA catabolism [3]. This may lower central fatigue and loss of motor coordination [60,76], determining sports performance. Finally, one can speculate that the beneficial regulation of NH_3_ concentration observed in our study may be related to combined supplementation and the synergistic impact of the preparations used. This suggests that the combined supplementation we used positively affected exercise adaptation, limited fatigue, and improved some metabolic processes, such as muscle protein synthesis (which was confirmed by the increase in FFM).

Our study has some limitations. First, we cannot be sure whether the athletes fully adhered to our recommendations. Second, more exercise characteristics than the level of aerobic capacity alone, e.g., an assessment of discipline-specific exercise abilities, would be desirable. However, in our athletic groups, some tests could not be performed due to potential interference with the training strategy. Third, it is also possible that the exercise time in high-intensity zones (submaximal and maximal) during the incremental treadmill test could be too short to reveal substantial changes in the supplementation-induced buffering capacity [20,38]. Fourth, Cr supplementation can lead to an increase in muscle hydration, which may affect FFM [10,11,77]. This should be taken into account when interpreting the results. However, in our opinion, the lack of changes in FFM in endurance athletes and the adherence to proper procedures for body composition measurement (DXA method) marginalized this limitation [78]. Fifth, there was no control/PLA group. On the other hand, it was not possible to apply a procedure in which athletes would only receive PLA or would not be supplemented for such a long period. Also, the recruitment of a control group would require the inclusion of athletes with lower training status, which would make it impossible to properly interpret the results. Finally, the small sample size may be a disadvantage. However, the strength of the study is that only highly-trained athletes were examined in two very homogenous groups, in contrast to many studies in which the effect of supplementation was assessed based on inactive or poorly trained people, making it difficult to transfer the results on competitive/professional athletes.

It should be emphasized that we studied the actual elite sprint and endurance athletes in real training conditions during a standard training cycle. We also implemented the crossover study design with a washout period. Although we have not demonstrated substantial changes, it must be underlined that even small differences in exercise indices can be beneficial for the competition results in elite sport. It is difficult to expect an enormous change in exercise adaptation in elite athletes because they already show a very high level of physical performance. Future research is needed to evaluate the effect of beta-alanine and extracellular alkaline agents in combination with other customary supplements used by sportsmen on exercise capacity and training adaptation.

## 5. Conclusions

The results of our study indicate that customarily used BCAA and Cr supplementation, combined with BA, seems to be more effective than BCAA and Cr supplementation combined with ALK as regards to improvements in fat-free mass and exercise adaptation. However, aerobic capacity did not improve. It also seems that the key factors, necessary for the supplementation effectiveness, are the discipline-specific exercise stimuli and the sports level of the athletes.

## Figures and Tables

**Table 1:** Baseline anthropometric, training, and diet characteristics of the studied athletes.

Variable		SPRINTERS	ENDURANCE	Sprinters vs. Endurance *
		Mean ± SD	Mean ± SD	*p*-Value
*n*		11	20	
Age	(years)	23.5 ± 3.4	22.3 ± 4.4	0.13
Body height	(cm)	184 ± 6	178 ± 7	0.04
Body mass	(kg)	77.8 ± 7.8	70.4 ± 11.3	0.01
Body mass index (BMI)	(kg·m^−2^)	23.0 ± 1.1	22.1 ± 2.3	0.29
Maximal oxygen uptake (VO_2_max)	(L·min^−1^)	4.15 ± 0.53	4.46 ± 0.82	0.27
Training experience	(years)	8.6 ± 2.5	8.6 ± 1.8	0.82
Energy intake	(kcal·day^−1^)	2922 ± 335	3179 ± 491	0.13
	(kcal·kg^−1^)	37.9 ± 5.3	46.6 ± 8.3	<0.01
Protein intake	(g·day^−1^)	129 ± 29	138 ± 34	0.48
	(g·kg^−1^)	1.7 ± 0.3	2.0 ± 0.5	0.052
	(% of energy)	17.8 ± 4.0	17.3 ± 3.3	0.68
Fat intake	(g·day^−1^)	102 ± 18	108 ± 28	0.50
	(g·kg^−1^)	1.3 ± 0.3	1.6 ± 0.5	0.09
	(% of energy)	31.4 ± 4.0	30.4 ± 4.6	0.55
Carbohydrate intake	(g·day^−1^)	378 ± 57	437 ± 63	0.02
	(g·kg^−1^)	4.9 ± 0.9	6.4 ± 1.1	<0.001
	(% of energy)	51.8 ± 4.4	55.3 ± 5.4	0.07

Values are expressed as the means ± standard deviation (SD). Abbreviations: BMI—body mass index and VO_2_max—maximal oxygen uptake. * *t*-tests or Mann–Whitney U tests for independent samples, depending on data distribution.

**Table 2:** Resting and exercise respiratory and aerobic capacity indices of sprinters and endurance athletes before and after supplementation.

Variable			Group BA-ALKpla_BCAA&TCM_				Group ALK-BApla_BCAA&TCM_			
			SPRINT		ENDURANCE		SPRINT		ENDURANCE	
			Mean ± SD	95% CI	Mean ± SD	95% CI	Mean ± SD	95% CI	Mean ± SD	95% CI
VE_rest_	(L∙min^−1^)	Pre	17.8 ± 2.9	15.8–19.8	16.1 ± 3.7	14.4–17.9	18.3 ± 2.2	16.8–19.8	16.1 ± 5.0	13.7–18.4
		Post	18.2 ± 2.2	16.7–19.7	16.7 ± 4.4	14.6–18.7	17.5 ± 2.0	16.1–18.9	16.5 ± 4.5	14.4–18.6
HR _rest_	(bpm)	Pre	88 ± 11	81–96	74 ± 16	67–82	90 ± 15	80–100	77 ± 16	69–84
		Post	86 ± 16	75–97	73 ± 12	68–79	85 ± 13	76–93	76 ± 20	67–85
VO_2__rest_	(L∙min^−1^)	Pre	0.55 ± 0.10	0.48–0.62	0.50 ± 0.12	0.44–0.55	0.57 ± 0.08	0.52–0.62	0.51 ± 0.15	0.43–0.58
		Post	0.58 ± 0.07	0.53–0.62	0.53 ± 0.15	0.46–0.60	0.59 ± 0.08	0.54–0.64	0.52 ± 0.13	0.45–0.58
RER _rest_		Pre	0.85 ± 0.08	0.79–0.90	0.86 ± 0.06	0.83–0.89	0.83 ± 0.07	0.78–0.88	0.85 ± 0.08	0.81–0.89
		Post	0.85 ± 0.07	0.80–0.89	0.83 ± 0.05	0.81–0.85	0.82 ± 0.03	0.80–0.84	0.85 ± 0.08	0.81–0.89
VE _VT_	(L∙min^−1^)	Pre	76.7 ± 19.9	63.4–90.1	77.0 ± 17.5	68.7–85.2	76.5 ± 18.7	64.0–89.1	76.9 ± 18.7	68.2–85.7
		Post	77.9 ± 22.1	63.0–92.7	79.3 ± 11.5	73.9–84.7	77.8 ± 18.2	65.5–90.0	81.5 ± 18.6	72.8–90.2
HR_VT_	(bpm)	Pre	156 ± 19	144–169	157 ± 13	151–163	159 ± 15	148–169	158 ± 11	153–163
		Post	15 ± 17	143–166	159 ± 14	153–166	156 ± 14	146–165	156 ± 8	152–160
VO_2__VT_	(L∙min^−1^)	Pre	2.86 ± 0.49	2.53–3.19	3.10 ± 0.66	2.80–3.41	2.95 ± 0.67	2.50–3.39	3.12 ± 0.69	2.80–3.45
		Post	2.85 ± 0.65	2.42–3.29	3.11 ± 0.70	2.05–3.59	2.90 ± 0.48	2.56–3.24	3.23 ± 0.72	2.89–3.56
RER_VT_		Pre	0.88 ± 0.06	0.84–0.92	0.90 ± 0.04	0.88–0.92	0.90 ± 0.04	0.87–0.92	0.99 ± 0.44	0.78–1.20
		Post	0.89 ± 0.07	0.84–0.94	0.90 ± 0.03	0.89–0.92	0.91 ± 0.03	0.88–0.93	0.89 ± 0.03	0.88–0.91
VE_MAX_	(L∙min^−1^)	Pre	152 ± 26	134–169	159 ± 23	149–170	154 ± 24	137–170	160 ± 25	148–172
		Post	153 ± 24	135–170	158 ± 23	148–169	152 ± 22	137–167	158 ± 26	146–170
HR_MAX_	(bpm)	Pre	192 ± 9	186–198	194 ± 10	189–198	193 ± 9	187–200	193 ± 10	188–198
		Post	193 ± 11	185–201	193 ± 10	188–197	192 ± 8	186–197	193 ± 11	188–198
VO_2__MAX_	(L∙min^−1^)	Pre	4.16 ± 0.60	3.76–4.56	4.45 ± 0.76	4.00–4.80	4.18 ± 0.53	3.82–4.53	4.55 ± 0.85	4.16–4.95
		Post	4.19 ± 0.54	3.80–4.57	4.45 ± 0.77	4.09–4.81	4.23 ± 0.51	3.89–4.57	4.51 ± 0.78	4.15–4.88
RER_MAX_		Pre	1.06 ± 0.03	1.03–1.08	1.09 ± 0.06	1.07–1.12	1.05 ± 0.03	1.04–1.07	1.09 ± 0.07	1.05–1.12
		Post	1.08 ± 0.04	1.05–1.10	1.09 ± 0.04	1.07–1.11	1.08 ± 0.02	1.06–1.10	1.07 ± 0.06	1.04–1.09

Data are the means ± standard deviation (SD) and 95% confidence intervals (CI). Abbreviations: ALK—Alkaline agents, BA—Beta-Alanine Carno Rush, BCAA—branched-chain amino acids, HR—heart rate, MAX—maximum values, VO_2_—oxygen uptake, VE—minute ventilation, VT—ventilatory threshold, pla—placebo, RER—respiratory exchange ratio, Rest—resting values, and TCM—creatine malate. No significant differences reported (*p* > 0.05).

## Supplementary Materials

The following materials are available online at https://www.mdpi.com/2072-6643/11/9/1961/s1: Table S1. A typical structure of training loads in sprinters and endurance athletes during two study periods. Table S2. Body mass and body composition of sprinters and endurance athletes before and after supplementation. Table S3. The level of selected biochemical markers in the blood in sprinters and endurance athletes before and after supplementation (resting, maximum, and recovery values).
