# Supplementary material for: The Effect of Beta-Alanine versus Alkaline Agent Supplementation Combined with Branched-Chain Amino Acids and Creatine Malate in Highly-Trained Sprinters and Endurance Athletes: A Randomized Double-Blind Crossover Study
Source: Nutrients. 2019 Aug 21;11(9):1961. doi: 10.3390/nu11091961 (PMC6769605; doi:10.3390/nu11091961)
Supplement: Supplementary file 1 [file nutrients-11-01961-s001.zip › Table S3_rev_nutrients-558695.docx]

**Table S3.** The level of selected biochemical markers in the blood of sprinters and endurance athletes before and after supplementation procedures (data for resting, maximum and restitution values).

|  |  | **Group BA-ALKpla_BCAA&TCM_** | | | | | | | | | | | | **Group ALK-BApla_BCAA&TCM_** | | | | | | | | | | | |
| --- | --- | --- | --- | --- | --- | --- | --- | --- | --- | --- | --- | --- | --- | --- | --- | --- | --- | --- | --- | --- | --- | --- | --- | --- | --- |
|  |  | **SPRINT** | | | | | | **ENDURANCE** | | | | | | **SPRINT** | | | | | | **ENDURANCE** | | | | | |
|  |  | Mean | ± | SD | 95% CI | | | Mean | ± | SD | 95% CI | | | Mean | ± | SD | 95% CI | | | Mean | ± | SD | 95% CI | | |
| **LArest (mmol·L^-1^)** | **Pre** | 1.3 | ± | 0.4 | 1.1 | - | 1.5 | 1.0 | ± | 0.3# | 0.9 | - | 1.1 | 1.4 | ± | 0.3* | 1.2 | - | 1.6 | 1.1 | ± | 0.3 | 1.0 | - | 1.2 |
|  | **Post** | 1.1 | ± | 0.3 | 0.8 | - | 1.3 | 1.0 | ± | 0.2†§ | 0.9 | - | 1.1 | 1.3 | ± | 0.3 | 1.1 | - | 1.5 | 1.0 | ± | 0.2‡ | 0.9 | - | 1.1 |
| **LAmax (mmol·L^-1^)** | **Pre** | 10.3 | ± | 1.1 | 9.6 | - | 11.0 | 10.7 | ± | 1.3 | 10.0 | - | 11.3 | 11.2 | ± | 1.7 | 10.1 | - | 12.3 | 10.5 | ± | 1.9 | 9.6 | - | 11.4 |
|  | **Post** | 9.6 | ± | 1.5 | 8.6 | - | 10.6 | 9.7 | ± | 1.4 | 9.1 | - | 10.4 | 10.7 | ± | 1.3 | 9.8 | - | 11.6 | 9.8 | ± | 1.6 | 9.1 | - | 10.5 |
| **LAR5 (mmol·L^-1^)** | **Pre** | 9.8 | ± | 1.5 | 8.9 | - | 10.8 | 9.6 | ± | 1.8 | 8.8 | - | 10.5 | 10.5 | ± | 1.9 | 9.3 | - | 11.8 | 9.5 | ± | 2.1 | 8.5 | - | 10.5 |
|  | **Post** | 9.1 | ± | 1.6 | 8.1 | - | 10.2 | 8.7 | ± | 1.3 | 8.0 | - | 9.3 | 10.2 | ± | 2.3 | 8.6 | - | 11.7 | 8.7 | ± | 1.6 | 7.9 | - | 9.5 |
| **LAR20 (mmol·L^-1^)** | **Pre** | 5.9 | ± | 1.5 | 4.9 | - | 6.9 | 6.0 | ± | 1.7 | 5.2 | - | 6.8 | 6.1 | ± | 1.9 | 4.8 | - | 7.3 | 6.1 | ± | 1.8 | 5.2 | - | 6.9 |
|  | **Post** | 4.9 | ± | 1.4 | 3.9 | - | 5.8 | 5.1 | ± | 1.3 | 4.4 | - | 5.7 | 6.1 | ± | 2.2 | 4.6 | - | 7.6 | 5.1 | ± | 1.6 | 4.3 | - | 5.8 |
| **LAR30 (mmol·L^-1^)** | **Pre** | 3.9 | ± | 1.2 | 3.1 | - | 4.7 | 4.3 | ± | 1.3 | 3.7 | - | 4.9 | 3.8 | ± | 1.9 | 2.5 | - | 5.1 | 4.2 | ± | 1.7 | 3.4 | - | 4.9 |
|  | **Post** | 3.1 | ± | 1.0 | 2.5 | - | 3.8 | 3.6 | ± | 1.0 | 3.1 | - | 4.0 | 4.0 | ± | 2.2 | 2.5 | - | 5.5 | 3.5 | ± | 1.2 | 2.9 | - | 4.0 |
| **NH_3_rest (µmol·L^-1^)** | **Pre** | 27.6 | ± | 2.8**^#^** | 25.7 | - | 29.5 | 21.9 | ± | 3.2 | 20.4 | - | 23.3 | 27.8 | ± | 3.4**^#^** | 25.6 | - | 30.1 | 22.0 | ± | 2.8 | 20.7 | - | 23.3 |
|  | **Post** | 25.9 | ± | 3.2^†^ | 23.8 | - | 28.0 | 20.8 | ± | 3.4 | 19.2 | - | 22.4 | 26.9 | ± | 3.5**^§^** | 24.6 | - | 29.3 | 21.4 | ± | 2.6 | 20.1 | - | 22.6 |
| **NH_3_max (µmol·L^-1^)** | **Pre** | 89.4 | ± | 8.0§ | 84.0 | - | 94.7 | 74.4 | ± | 8.4 | 70.4 | - | 78.3 | 84.6 | ± | 7.8^&^ | 79.4 | - | 89.9 | 73.5 | ± | 10.3**^‡‡^** | 68.6 | - | 78.3 |
|  | **Post** | 80.4 | ± | 7.7* | 75.2 | - | 85.5 | 68.9 | ± | 8.3^*^ | 65.0 | - | 72.8 | 82.4 | ± | 8.4**^&^** | 76.7 | - | 88.0 | 70.2 | ± | 9.2**^‡^** | 65.8 | - | 74.5 |
| **NH_3_R5 (µmol/L)** | **Pre** | 77.3 | ± | 7.2^¶^ | 72.5 | - | 82.1 | 64.2 | ± | 10.4 | 59.3 | - | 69.1 | 72.5 | ± | 6.8**^‡^** ^‡‡^ | 68.0 | - | 77.1 | 62.6 | ± | 9.0 | 58.4 | - | 66.7 |
|  | **Post** | 70.9 | ± | 6.7^‡‡^ | 66.4 | - | 75.4 | 59.3 | ± | 11.0^**^ | 54.1 | - | 64.4 | 69.9 | ± | 6.0^**^ | 65.9 | - | 73.9 | 60.4 | ± | 8.5 | 56.4 | - | 64.3 |
| **NH_3_R20 (µmol·L^-1^)** | **Pre** | 58.3 | ± | 7.8§ | 53.0 | - | 63.5 | 45.4 | ± | 6.1 | 42.5 | - | 48.2 | 53.5 | ± | 7.6^Ω@^ | 48.4 | - | 58.7 | 45.7 | ± | 7.2 | 42.3 | - | 49.1 |
|  | **Post** | 54.4 | ± | 7.4 | 49.4 | - | 59.3 | 41.2 | ± | 5.8**^*^** | 38.5 | - | 43.8 | 51.3 | ± | 8.0^Φ‡‡^ | 45.9 | - | 56.6 | 43.6 | ± | 6.5 | 40.5 | - | 46.6 |
| **NH_3_R30 (µmol·L^-1^)** | **Pre** | 43.2 | ± | 5.4**^‡‡^**^‽^**^#^** | 39.5 | - | 46.8 | 35.1 | ± | 3.9 | 33.2 | - | 36.9 | 38.0 | ± | 4.4^€ Ω^ | 35.0 | - | 41.0 | 34.2 | ± | 3.7**^‡‡^** | 32.4 | - | 35.9 |
|  | **Post** | 39.1 | ± | 5.0 ^Ω^ | 35.7 | - | 42.4 | 31.2 | ± | 3.9**^*^** | 29.3 | - | 33.0 | 36.6 | ± | 4.1**^₸^** ^Ω^ | 33.9 | - | 39.4 | 33.9 | ± | 4.2**^©^** | 31.9 | - | 35.8 |
| **WBCrest (109·L^-1^)** | **Pre** | 5.55 | ± | 1.17 | 4.76 | - | 6.33 | 5.13 | ± | 1.06 | 4.63 | - | 5.62 | 6.04 | ± | 2.53 | 4.34 | - | 7.74 | 5.05 | ± | 1.19 | 4.49 | - | 5.61 |
|  | **Post** | 5.20 | ± | 1.50 | 4.19 | - | 6.21 | 4.98 | ± | 1.11 | 4.46 | - | 5.50 | 4.95 | ± | 0.97 | 4.29 | - | 5.60 | 4.65 | ± | 1.04 | 4.16 | - | 5.13 |
| **Lym_rest_ (109·L^-1^)** | **Pre** | 1.72 | ± | 0.31 | 1.51 | - | 1.93 | 1.74 | ± | 0.41 | 1.55 | - | 1.93 | 1.74 | ± | 0.29 | 1.54 | - | 1.93 | 1.76 | ± | 0.46 | 1.54 | - | 1.97 |
|  | **Post** | 1.63 | ± | 0.40 | 1.36 | - | 1.90 | 1.81 | ± | 0.37 | 1.63 | - | 1.98 | 1.42 | ± | 0.23 | 1.27 | - | 1.57 | 1.62 | ± | 0.29 | 1.48 | - | 1.76 |
| **Mon_rest_ (109·L^-1^)** | **Pre** | 0.33 | ± | 0.10 | 0.26 | - | 0.40 | 0.37 | ± | 0.09 | 0.32 | - | 0.41 | 0.37 | ± | 0.11 | 0.30 | - | 0.45 | 0.34 | ± | 0.13 | 0.28 | - | 0.40 |
|  | **Post** | 0.45 | ± | 0.27 | 0.28 | - | 0.63 | 0.44 | ± | 0.16 | 0.36 | - | 0.51 | 0.30 | ± | 0.09 | 0.24 | - | 0.36 | 0.37 | ± | 0.11 | 0.31 | - | 0.42 |
| **Gra_rest_ (109·L^-1^)** | **Pre** | 3.50 | ± | 1.13 | 2.74 | - | 4.26 | 3.01 | ± | 0.92 | 2.58 | - | 3.44 | 3.94 | ± | 2.30 | 2.39 | - | 5.48 | 2.91 | ± | 1.08 | 2.40 | - | 3.41 |
|  | **Post** | 3.13 | ± | 1.07 | 2.41 | - | 3.84 | 2.75 | ± | 0.90 | 2.33 | - | 3.17 | 3.25 | ± | 0.86 | 2.67 | - | 3.82 | 2.63 | ± | 0.93 | 2.20 | - | 3.07 |
| **Lym%_rest_** | **Pre** | 32.0 | ± | 7.8 | 26.7 | - | 37.2 | 34.8 | ± | 7.3 | 31.3 | - | 38.2 | 32.0 | ± | 10.0 | 25.3 | - | 38.8 | 36.2 | ± | 8.6 | 32.2 | - | 40.2 |
|  | **Post** | 32.6 | ± | 7.8 | 27.4 | - | 37.8 | 37.0 | ± | 8.1 | 33.2 | - | 40.8 | 29.3 | ± | 6.3 | 25.1 | - | 33.5 | 35.8 | ± | 8.2 | 32.0 | - | 39.6 |
| **Mon%_rest_** | **Pre** | 6.28 | ± | 2.15 | 4.84 | - | 7.73 | 7.30 | ± | 1.33 | 6.68 | - | 7.92 | 6.12 | ± | 2.16^Δ^**^@©^** | 4.66 | - | 7.57 | 6.70 | ± | 1.83**^‡‡^** | 5.84 | - | 7.55 |
|  | **Post** | 8.06 | ± | 2.51^€‡‡^**^@^** | 6.38 | - | 9.75 | 8.63 | ± | 1.93 | 7.72 | - | 9.54 | 6.48 | ± | 1.25 | 5.64 | - | 7.32 | 8.10 | ± | 1.71^**^ | 7.30 | - | 8.90 |
| **Gra%_rest_** | **Pre** | 61.7 | ± | 8.4 | 56.1 | - | 67.4 | 57.9 | ± | 7.9 | 54.2 | - | 61.6 | 61.9 | ± | 11.7 | 54.0 | - | 69.7 | 57.1 | ± | 9.5 | 52.6 | - | 61.5 |
|  | **Post** | 59.3 | ± | 8.3 | 53.8 | - | 64.9 | 54.3 | ± | 8.7 | 50.3 | - | 58.4 | 64.1 | ± | 7.0 | 59.4 | - | 68.8 | 56.1 | ± | 8.7 | 52.0 | - | 60.2 |
| **RB _rest_ (1012·L^-1^)** | **Pre** | 4.80 | ± | 0.36 | 4.55 | - | 5.04 | 4.79 | ± | 0.43 | 4.58 | - | 4.99 | 4.91 | ± | 0.38 | 4.66 | - | 5.16 | 4.77 | ± | 0.38 | 4.59 | - | 4.95 |
|  | **Post** | 4.87 | ± | 0.33 | 4.65 | - | 5.09 | 4.67 | ± | 0.34 | 4.51 | - | 4.83 | 4.78 | ± | 0.25 | 4.62 | - | 4.95 | 4.59 | ± | 0.42 | 4.40 | - | 4.79 |
| **Hb_rest_ (mmol·L^-1^)** | **Pre** | 8.67 | ± | 0.56 | 8.29 | - | 9.05 | 8.77 | ± | 0.71 | 8.44 | - | 9.10 | 8.85 | ± | 0.69 | 8.38 | - | 9.31 | 8.77 | ± | 0.69 | 8.44 | - | 9.09 |
|  | **Post** | 8.80 | ± | 0.59 | 8.40 | - | 9.19 | 8.50 | ± | 0.51 | 8.26 | - | 8.74 | 8.77 | ± | 0.41 | 8.50 | - | 9.04 | 8.43 | ± | 0.76 | 8.07 | - | 8.79 |
| **Ht_rest_ (L·L^-1^)** | **Pre** | 0.41 | ± | 0.03 | 0.39 | - | 0.43 | 0.41 | ± | 0.03 | 0.40 | - | 0.43 | 0.42 | ± | 0.03 | 0.40 | - | 0.44 | 0.41 | ± | 0.03 | 0.40 | - | 0.42 |
|  | **Post** | 0.42 | ± | 0.03 | 0.40 | - | 0.44 | 0.41 | ± | 0.03 | 0.39 | - | 0.42 | 0.42 | ± | 0.02 | 0.41 | - | 0.43 | 0.40 | ± | 0.03 | 0.39 | - | 0.42 |
| **MCV_rest_ (fL)** | **Pre** | 85.7 | ± | 3.8 | 83.2 | - | 88.3 | 86.4 | ± | 4.4 | 84.4 | - | 88.4 | 85.6 | ± | 3.4 | 83.4 | - | 87.9 | 86.4 | ± | 5.0 | 84.1 | - | 88.8 |
|  | **Post** | 86.2 | ± | 2.4 | 84.6 | - | 87.8 | 87.5 | ± | 3.8 | 85.7 | - | 89.3 | 87.7 | ± | 2.9 | 85.7 | - | 89.6 | 88.2 | ± | 3.5 | 86.6 | - | 89.9 |
| **MCH_rest_ (fmol)** | **Pre** | 1.81 | ± | 0.08 | 1.76 | - | 1.87 | 1.84 | ± | 0.10 | 1.79 | - | 1.89 | 1.80 | ± | 0.07 | 1.76 | - | 1.85 | 1.84 | ± | 0.11 | 1.79 | - | 1.89 |
|  | **Post** | 1.81 | ± | 0.07 | 1.76 | - | 1.86 | 1.82 | ± | 0.11 | 1.77 | - | 1.87 | 1.84 | ± | 0.07 | 1.79 | - | 1.89 | 1.84 | ± | 0.09 | 1.79 | - | 1.88 |
| **MCHC_rest_ (mmol·L^-1^)** | **Pre** | 21.1 | ± | 0.3 | 20.9 | - | 21.3 | 21.3 | ± | 0.6 | 21.0 | - | 21.6 | 21.0 | ± | 0.5 | 20.7 | - | 21.3 | 21.3 | ± | 0.8 | 21.0 | - | 21.7 |
|  | **Post** | 21.0 | ± | 0.6 | 20.6 | - | 21.4 | 20.8 | ± | 0.8 | 20.5 | - | 21.2 | 20.9 | ± | 0.5 | 20.6 | - | 21.3 | 20.8 | ± | 0.8 | 20.5 | - | 21.2 |
| **RDW_rest_ (%)** | **Pre** | 11.1 | ± | 0.5 | 10.7 | - | 11.5 | 11.6 | ± | 1.1 | 11.1 | - | 12.1 | 11.2 | ± | 0.6 | 10.8 | - | 11.6 | 11.8 | ± | 1.2**^‡‡^** | 11.3 | - | 12.4 |
|  | **Post** | 11.1 | ± | 0.5 | 10.8 | - | 11.5 | 11.2 | ± | 0.5 | 10.9 | - | 11.4 | 11.0 | ± | 0.4 | 10.7 | - | 11.2 | 11.4 | ± | 0.7 | 11.0 | - | 11.7 |
| **Plt_rest_ (109·L^-1^)** | **Pre** | 213 | ± | 42 | 185 | - | 241 | 204 | ± | 62 | 175 | - | 234 | 210 | ± | 36 | 186 | - | 234 | 200 | ± | 74 | 166 | - | 235 |
|  | **Post** | 222 | ± | 34 | 199 | - | 245 | 192 | ± | 67 | 161 | - | 223 | 219 | ± | 35 | 195 | - | 242 | 187 | ± | 47 | 165 | - | 209 |
| **MPV_rest_ (fL)** | **Pre** | 8.99 | ± | 0.65 | 8.56 | - | 9.43 | 9.16 | ± | 0.85 | 8.76 | - | 9.55 | 8.87 | ± | 0.74 | 8.38 | - | 9.37 | 9.17 | ± | 0.87 | 8.76 | - | 9.58 |
|  | **Post** | 9.10 | ± | 0.90 | 8.49 | - | 9.71 | 9.20 | ± | 0.85 | 8.80 | - | 9.60 | 9.06 | ± | 0.68 | 8.60 | - | 9.52 | 9.26 | ± | 0.94 | 8.82 | - | 9.70 |
| **PCT_rest_ (cL·L^-1^)** | **Pre** | 0.19 | ± | 0.03 | 0.17 | - | 0.21 | 0.18 | ± | 0.04 | 0.16 | - | 0.20 | 0.18 | ± | 0.02 | 0.17 | - | 0.20 | 0.18 | ± | 0.06 | 0.15 | - | 0.21 |
|  | **Post** | 0.20 | ± | 0.02 | 0.19 | - | 0.21 | 0.17 | ± | 0.05 | 0.15 | - | 0.20 | 0.20 | ± | 0.03 | 0.18 | - | 0.21 | 0.17 | ± | 0.04 | 0.15 | - | 0.19 |
| **PDW_rest_ (%)** | **Pre** | 14.2 | ± | 1.1**^©^** | 13.5 | - | 15.0 | 13.1 | ± | 1.0 | 12.7 | - | 13.6 | 13.4 | ± | 1.1 | 12.7 | - | 14.2 | 13.6 | ± | 1.1 | 13.1 | - | 14.1 |
|  | **Post** | 13.6 | ± | 0.7 | 13.1 | - | 14.1 | 12.8 | ± | 1.4 | 12.2 | - | 13.5 | 13.6 | ± | 0.9 | 12.9 | - | 14.2 | 13.1 | ± | 1.1 | 12.5 | - | 13.6 |

Data are mean ± standard deviation (SD) and 95% confidence intervals (CI). Abbreviations: 0–resting value, ALK–Alkaline agents, BA–Beta-Alanine Carno Rush, BCAA–branched-chain amino acids, Gra–granulocytes, LA–lactate, Hb–hemoglobin, Ht–hematocrit, Lym– lymphocytes, MCH–mean corpuscular hemoglobin, MCHC–mean corpuscular hemoglobin concentration, MCV–mean corpuscular volume, Mon–monocytes, MPV– mean platelet volume, NH–ammonia, pla–placebo, PCT–plateletcrit, PDW–platelet distribution width, Plt–platelets, R–post-exercise recovery period, RBC–red blood cells, RDW–red blood cell distribution width, TCM–creatine malate, WBC–white blood cells. *p < 0.001, **p<0.02; *** p<0.05: Significantly different from PRE value; ^#^p < 0.001, ^§^p < 0.005; ^¶^p < 0.02: Significantly different from endurance group for all periods; ^†^p < 0.05: Significantly different from endurance group for BA-ALKpla_BCAA&TCM_ periods and Post ALK-BApla_BCAA&TCM_; ^&^p < 0.02: Significantly different from PRE value of endurance group; ‡p < 0.05; Δp<0.001: Significantly different from endurance group for PRE BA-ALKpla_BCAA&TCM_ period; ^Ω^p < 0.001; ^‡‡^p < 0.02; ^©^p < 0.05: Significantly different from endurance group for POST BA-ALKpla_BCAA&TCM_ period; ^₸^p < 0.001; ^Φ^p < 0.01; ^€^p < 0.05: Significantly different from sprint group for PRE BA-ALKpla_BCAA&TCM_ period; ^‽^p < 0.005; ^@^p<0.05: Significantly different from endurance group for POST ALK-BApla_BCAA&TCM_ period.
