# Supplementary material for: The Effect of Beta-Alanine versus Alkaline Agent Supplementation Combined with Branched-Chain Amino Acids and Creatine Malate in Highly-Trained Sprinters and Endurance Athletes: A Randomized Double-Blind Crossover Study
Source: Nutrients. 2019 Aug 21;11(9):1961. doi: 10.3390/nu11091961 (PMC6769605; doi:10.3390/nu11091961)
Supplement: Supplementary file 1 [file nutrients-11-01961-s001.zip › Table S1_rev_nutrients-558695.docx]

**Table S1.** Typical structure of training loads in tested sprinters and endurances between successive examination.

|  | 1^st^ 8 weeks supplementation | | 2^nd^ 8 weeks supplementation | |
| --- | --- | --- | --- | --- |
|  | Sprinters | Endurances | Sprinters | Endurances |
| Training sessions (no.) | 46 | 85 | 47 | 83 |
| Strength training sessions (no.) | 16 | 8 | 16 | 8 |
| Competitions (no.) | - | - | 5 | 2 |
| Net exercise time (hours) |  |  |  |  |
| total | 51.6 | 108.2 | 52.1 | 101.1 |
| per one training session | 1.12 | 1.27 | 1.11 | 1.21 |
| Exercise zones (% of total time) |  |  |  |  |
| aerobic compensation | 61.2 | 58.2 | 65.4 | 58.4 |
| aerobic stimulation | 12.4 | 23.7 | 10.4 | 24.2 |
| aerobic-anaerobic | 16.7 | 14.0 | 14.1 | 12.6 |
| anaerobic lactacid | 4.1 | 2.9 | 5.9 | 3.1 |
| anerobic non-lactacid | 5.6 | 1.2 | 4.2 | 1.7 |
