# Supplementary material for: The Effect of Beta-Alanine versus Alkaline Agent Supplementation Combined with Branched-Chain Amino Acids and Creatine Malate in Highly-Trained Sprinters and Endurance Athletes: A Randomized Double-Blind Crossover Study
Source: Nutrients. 2019 Aug 21;11(9):1961. doi: 10.3390/nu11091961 (PMC6769605; doi:10.3390/nu11091961)
Supplement: Supplementary file 1 [file nutrients-11-01961-s001.zip › Table S2_rev_nutrients-558695.docx]

**Table S2.** Body mass and detailed body composition of sprinters and endurance athletes before and after supplementation procedures.

|  |  | **Group BA-ALKpla_BCAA&TCM_** | | | | | | | | | | | | **Group ALK-BApla_BCAA&TCM_** | | | | | | | | | | | |
| --- | --- | --- | --- | --- | --- | --- | --- | --- | --- | --- | --- | --- | --- | --- | --- | --- | --- | --- | --- | --- | --- | --- | --- | --- | --- |
|  |  | **SPRINT** | | | | | | **ENDURANCE** | | | | | | **SPRINT** | | | | | | **ENDURANCE** | | | | | |
|  |  | Mean | ± | SD | 95% CI | | | Mean | ± | SD | 95% CI | | | Mean | ± | SD | 95% CI | | | Mean | ± | SD | 95% CI | | |
| **Body mass (kg)** | **Pre** | 77.8 | ± | 7.4 | 72.8 | - | 82.7 | 70.4 | ± | 8.9 | 66.2 | - | 74.5 | 78.2 | ± | 7.4 | 73.3 | - | 83.2 | 70.7 | ± | 9.8 | 66.1 | - | 75.3 |
|  | **Post** | 79.0 | ± | 7.8 | 73.8 | - | 84.3 | 70.3 | ± | 8.7 | 66.2 | - | 74.3 | 78.5 | ± | 7.2 | 73.7 | - | 83.4 | 70.2 | ± | 9.1 | 65.9 | - | 74.4 |
| **Body Mass Index (kg·m^-2^)** | **Pre** | 23.0 | ± | 1.1 | 22.2 | - | 23.7 | 22.1 | ± | 2.2 | 21.1 | - | 23.1 | 23.1 | ± | 1.2 | 22.4 | - | 23.9 | 22.2 | ± | 2.4 | 21.1 | - | 23.3 |
|  | **Post** | 23.3 | ± | 1.1 | 22.6 | - | 24.0 | 22.1 | ± | 2.2 | 21.0 | - | 23.1 | 23.2 | ± | 1.1 | 22.4 | - | 24.0 | 22.0 | ± | 2.3 | 20.9 | - | 23.0 |
| **Total Bone Mass (kg)** | **Pre** | 3.47 | ± | 0.43 | 3.18 | - | 3.76 | 3.02 | ± | 0.44 | 2.81 | - | 3.23 | 3.48 | ± | 0.44 | 3.18 | - | 3.77 | 3.02 | ± | 0.44 | 2.82 | - | 3.23 |
|  | **Post** | 3.49 | ± | 0.43 | 3.20 | - | 3.77 | 3.03 | ± | 0.45 | 2.82 | - | 3.24 | 3.47 | ± | 0.43 | 3.19 | - | 3.76 | 3.03 | ± | 0.45 | 2.82 | - | 3.24 |
| **Total Fat Mass (kg)** | **Pre** | 10.0 | ± | 2.3 | 8.4 | - | 11.5 | 11.5 | ± | 3.2 | 10.0 | - | 13.0 | 10.2 | ± | 2.5 | 8.5 | - | 11.9 | 11.4 | ± | 2.8 | 10.1 | - | 12.7 |
|  | **Post** | 9.5 | ± | 2.0 | 8.2 | - | 10.9 | 11.1 | ± | 2.8 | 9.7 | - | 12.4 | 9.4 | ± | 2.3 | 7.8 | - | 10.9 | 10.8 | ± | 3.0 | 9.4 | - | 12.2 |
| **Total TissueFat (%)** | **Pre** | 13.5 | ± | 4.4 | 10.6 | - | 16.4 | 17.0 | ± | 4.9 | 14.7 | - | 19.3 | 13.7 | ± | 4.8 | 10.5 | - | 16.9 | 16.8 | ± | 4.1 | 14.9 | - | 18.8 |
|  | **Post** | 12.7 | ± | 3.8 | 10.1 | - | 15.3 | 16.5 | ± | 4.6 | 14.4 | - | 18.7 | 12.9 | ± | 4.2 | 10.1 | - | 15.7 | 16.1 | ± | 4.6 | 13.9 | - | 18.3 |
| **Legs Fat-Free Mass (kg)** | **Pre** | 24.4 | ± | 3.2 | 22.3 | - | 26.6 | 20.3 | ± | 3.2 | 18.8 | - | 21.8 | 24.7 | ± | 3.6 | 22.3 | - | 27.1 | 20.7 | ± | 3.5 | 19.0 | - | 22.3 |
|  | **Post** | 25.7 | ± | 4.1*^&^ | 22.9 | - | 28.4 | 20.5 | ± | 3.0 | 19.1 | - | 21.9 | 25.1 | ± | 3.3**^‡^** | 22.9 | - | 27.3 | 20.6 | ± | 3.1 | 19.1 | - | 22.1 |
| **Gynoid Fat-Free Mass (kg)** | **Pre** | 11.0 | ± | 1.4 | 10.1 | - | 12.0 | 9.2 | ± | 1.5 | 8.5 | - | 9.9 | 11.3 | ± | 1.5**^‡^** | 10.3 | - | 12.3 | 9.4 | ± | 1.5 | 8.7 | - | 10.1 |
|  | **Post** | 11.5 | ± | 1.5**^†&^** | 10.5 | - | 12.5 | 9.3 | ± | 1.5 | 8.6 | - | 10.0 | 11.4 | ± | 1.5**^&^** | 10.4 | - | 12.4 | 9.4 | ± | 1.5 | 8.7 | - | 10.1 |
| **Total Fat-Free Mass (kg)** | **Pre** | 68.7 | ± | 8.9 | 62.7 | - | 74.7 | 59.5 | ± | 8.9 | 55.3 | - | 63.6 | 69.0 | ± | 8.9 | 63.0 | - | 75.0 | 59.9 | ± | 9.1 | 55.6 | - | 64.1 |
|  | **Post** | 70.4 | ± | 9.1****** | 64.3 | - | 76.5 | 59.7 | ± | 8.8 | 55.6 | - | 63.8 | 69.5 | ± | 8.8 | 63.6 | - | 75.5 | 59.9 | ± | 8.7 | 55.8 | - | 64.0 |

Data are mean ± standard deviation (SD) and 95% confidence intervals (CI). Abbreviations: ALK–Alkaline agents, BA–Beta-Alanine Carno Rush, BCAA–branched-chain amino acids, pla–placebo, TCM–creatine malate. *p < 0.001, †p < 0.003, **p < 0.01: Significantly different from PRE value; ^&^p < 0.02, ^‡^p < 0.05: Significantly different from endurance group for all periods.
